# The Treatment of Gout by Hot Dry Air

**Published:** 1897-07-17

**Authors:** 


### Medical Progress and Hospital Clinics.

[*The Editor will be glad to receive offers of co-operation and contributions from members of the profession. All letters should be addressed to* The Editor, at the Office, 28 & 29, Southampton Street, Strand, London, W.C.]

### THE TREATMENT OF GOUT BY HOT DRY AIR.

It is a matter of some interest to compare the local use of hot dry air with its application to the whole of the body as in the Turkish bath. A good deal has lately been done in the treatment of rheumatic and gouty joints by the local application of hot air, and the reports as to its power of relieving pain and removing stiffness are very encouraging. According to Dr. Sibley, the effect of exposing a limb to a temperature of up to 300 deg. F. for from half an hour to an hour is that first the limb treated and then the whole body breaks out into a profuse perspiration, while the pulse and to a lesser degree the respirations are increased in frequency. Even the general temperature of the body is increased, as is shown, by a rise of one or two degrees in the thermometer placed in the mouth. These are, in fact, just the same results as take place in the Turkish bath, with the exception that in the bath there is no special incidence of the perspiration on the affected limb.

It is probable that the beneficial effect of the local application of dry heat in cases of gout, whether acute or chronic, occurs in two or even in three ways which are quite distinct from one another. Everyone knows how greatly the pain in such cases is relieved for a time by the application of heat in any form. In the case of moist heat this is no doubt largely due to mere vascular relaxation, the excess of heat having to be carried off by passing an increased supply of blood through the part, although the sedative effect of moderate heat upon the sensory nerves also has its share in the effect.

But with hot dry air the limb protects itself from becoming overheated by the production of profuse perspirations, as well as by the transference of heat, viâ the blood-vessels, to other parts of the body, so that in the application of heat in this form not only do the vessels become largely dilated and the tissues flooded with blood, but a considerable local emunctory effect is produced by the excessive local perspiration. If, however, the heat be continued any length of time local evaporation, however dry the air be kept, is not sufficient to keep down the temperature, and a general perspiration occurs such as takes place in a Turkish bath. In this way the undoubted good effect of the Turkish bath, which acts as a powerful stimulant to general perspiration, is also in some degree obtained even by a local application of heat. It is clear then that the local application of hot dry air does good in directions which are common on the one hand to the local application of moist heat, and on the other to the general application of hot air. To what then are we to attribute the added efficacy of the local applications of hot, dry air to which Dr. Sibley and others bear such strong testimony. The probabilities are that the greater efficacy of the local over the generalised application of this form of heat is due to the fact that the heart is not strong enough, nor, in fact, is the quantity of blood in the body great enough to produce such perspiration as to enable the whole body to withstand for long such temperatures as can safely be applied to localised portions. A higher temperature can be applied and a more profuse perspiration can be maintained, and can be kept up for a much longer time, locally than could be borne without inducing faintness if applied to the whole surface. It is then to the very great vascular dilation, and to the very large local excretion of perspiration which is produced, that the local application of dry air owes its peculiar efficacy, in addition to which it seems to possess the same soothing effects as moist heat, and to some extent the general eliminative effect of the Turkish bath. Some of the cases related are very striking.

